# Supplementary material for: Identification and functional analysis of non-coding regulatory small RNA FenSr3 in Bacillus amyloliquefaciens LPB-18
Source: PeerJ. 2023 May 15;11:e15236. doi: 10.7717/peerj.15236 (PMC10194069; doi:10.7717/peerj.15236)
Supplement: Supplemental Information 4 [file peerj-11-15236-s004.zip › KO/CK-vs-T1_map/map04657.html]

KEGG PATHWAY: IL-17 signaling pathway - Reference pathway


|  |  |
| --- | --- |
| **IL-17 signaling pathway - Reference pathway** |  |

[
Pathway menu
| Organism menu
| Pathway entry
| Show description
| User data mapping
]

The interleukin 17 (IL-17) family, a subset of cytokines consisting of IL-17A-F, plays crucial roles in both acute and chronic inflammatory responses. IL-17A, the hallmark cytokine of the newly defined T helper 17 (TH17) cell subset, has important roles in protecting the host against extracellular pathogens, but also promotes inflammatory pathology in autoimmune disease, whereas IL-17F is mainly involved in mucosal host defense mechanisms. IL-17E (IL-25) is an amplifier of Th2 immune responses. IL-17C has biological functions similar to those of IL-17A. The functions of IL-17B and IL-17D remain largely elusive. The IL-17 family signals via their correspondent receptors and activates downstream pathways that include NF-kappaB, MAPKs and C/EBPs to induce the expression of antimicrobial peptides, cytokines and chemokines. The receptor proximal adaptor Act1 (an NF-kappaB activator 1) is considered as the master mediator in IL-17A signaling. It is likely that Act1 is a common signal adaptor also shared by other members mediated signalings in this family.

|  |  |  |
| --- | --- | --- |
| Reference pathway | 184% 150% 122% 100% 82% 67% 55% | 图片下载 |
